# Tick-Host Range Adaptation: Changes in Protein Profiles in Unfed Adult *Ixodes scapularis* and *Amblyomma americanum* Saliva Stimulated to Feed on Different Hosts

**DOI:** 10.3389/fcimb.2017.00517

**Published:** 2017-12-19

**Authors:** Lucas Tirloni, Tae K. Kim, Antônio F. M. Pinto, John R. Yates, Itabajara da Silva Vaz, Albert Mulenga

**Affiliations:** ^1^Department of Veterinary Pathobiology, College of Veterinary Medicine, Texas A&M University, College Station, TX, United States; ^2^Centro de Biotecnologia, Universidade Federal do Rio Grande do Sul, Porto Alegre, Brazil; ^3^Mass Spectrometry Center, The Salk Institute for Biological Studies, La Jolla, CA, United States; ^4^Department of Chemical Physiology, The Scripps Research Institute, La Jolla, CA, United States; ^5^Faculdade de Veterinária, Universidade Federal do Rio Grande do Sul, Porto Alegre, Brazil

**Keywords:** tick, saliva, proteomic, tick-host relationship, host adaptation

## Abstract

Understanding the molecular basis of how ticks adapt to feed on different animal hosts is central to understanding tick and tick-borne disease (TBD) epidemiology. There is evidence that ticks differentially express specific sets of genes when stimulated to start feeding. This study was initiated to investigate if ticks such as *Ixodes scapularis* and *Amblyomma americanum* that are adapted to feed on multiple hosts utilized the same sets of proteins to prepare for feeding. We exposed *I. scapularis* and *A. americanum* to feeding stimuli of different hosts (rabbit, human, and dog) by keeping unfed adult ticks enclosed in a perforated microfuge in close contact with host skin, but not allowing ticks to attach on host. Our data suggest that ticks of the same species differentially express tick saliva proteins (TSPs) when stimulated to start feeding on different hosts. SDS-PAGE and silver staining analysis revealed unique electrophoretic profiles in saliva of *I. scapularis* and *A. americanum* that were stimulated to feed on different hosts: rabbit, human, and dog. LC-MS/MS sequencing and pairwise analysis demonstrated that *I. scapularis* and *A. americanum* ticks expressed unique protein profiles in their saliva when stimulated to start feeding on different hosts: rabbit, dog, or human. Specifically, our data revealed TSPs that were unique to each treatment and those that were shared between treatments. Overall, we identified a total of 276 and 340 non-redundant *I. scapularis* and *A. americanum* TSPs, which we have classified into 28 functional classes including: secreted conserved proteins (unknown functions), proteinase inhibitors, lipocalins, extracellular matrix/cell adhesion, heme/iron metabolism, signal transduction and immunity-related proteins being the most predominant in saliva of unfed ticks. With exception of research on vaccines against *Rhipicephalus microplus*, which its natural host, cattle, research on vaccine against other ticks relies feeding ticks on laboratory animals. Data here suggest that relying on lab animal tick feeding data to select target antigens could result in prioritizing irrelevant anti-tick vaccine targets that are expressed when ticks feed on laboratory animals. This study provides the platform that could be utilized to identify relevant target anti-tick vaccine antigens, and will facilitate early stage tick feeding research.

## Introduction

Ticks and tick-borne diseases (TBD) cause significant problems to global and veterinary health, impacting huge losses in the livestock industry (Jongejan and Uilenberg, 2004; Grisi et al., 2014). Their impact on public health has been on a steady climb since the 1980s (Dantas-Torres et al., 2012). In absence of vaccines against TBD agents, controlling of ticks using acaricides is the only reliable method to prevent human and animal TBD infections. Limitations of acaricide-based tick control have necessitated the search for alternative tick control methods (Domingos et al., 2013; Abbas et al., 2014) in which immunization of animals against tick feeding has been advocated as a sustainable alternative (de la Fuente and Kocan, 2006; de la Fuente et al., 2007; Parizi et al., 2012). Without the ability to attach and feed on its host, ticks cannot cause skin damage nor transmit TBD agents. Thus, a deeper understanding of tick feeding is needed as a mean to find molecular targets that can be useful for development of novel tick control methods. From this perspective, tick-feeding physiology continues to receive significant research attention.

Fundamental organismal level research has documented a series of tick behavioral and physiological changes through which the tick proceeds to successfully feed and transmit disease agents. Other lines of research have attempted to identify molecular mechanisms underlying tick feeding behavioral and physiological changes leading to successful tick feeding (Nuttall and Labuda, 2004; Mulenga et al., 2007). In this way, molecular targets for innovative tick control could be discovered. We are interested in understanding the molecular basis of how the tick adapts to feed on different animal hosts. The tick's adaptation to feed on different animal species is central to TBD epidemiology. Medically important tick species such as *Amblyomma americanum* and *Ixodes scapularis* that transmit a combined 11 of the 16 human TBD agents in the USA are effective vectors (US Centers for Disease Control and Prevention—CDC, https://www.cdc.gov/ticks/diseases/index.html) because they can feed on multiple hosts including humans (Dantas-Torres et al., 2012). Ticks acquire TBD agents from wild animal reservoirs and transmit to the human population. Likewise, the causative agents of economically important animal diseases such as *Ehrlichia ruminantium* and *Theileria parva* are transferred from wildlife reservoirs to domestic animal population due to the ability of the tick vector to feed on different animal species (van Vuuren and Penzhorn, 2015). The southern cattle fever tick, *Rhipicephalus microplus*, is specialized to feed on cattle, however it may also feed on white tailed deer and other deer species which maintains the tick population in the environment in the absence of cattle (Duarte Cancado et al., 2009), although ticks that feed on deer have a lower fitness (Popara et al., 2013). Likewise, *Rhipicephalus sanguineus*, specialized to feed on dogs can also feed on humans (Dantas-Torres et al., 2006; Dantas-Torres, 2010), in which this tick is capable of transmitting *Rickettsia rickettssii* from dogs to humans in areas where the principal vector ticks *Dermacentor variabilis* and *Dermacentor andersoni* are absent (Piranda et al., 2011; Drexler et al., 2014). Despite its importance, the molecular basis of how the tick adapts to feed on different hosts remains poorly understood.

Ticks are pool feeders, and accomplish feeding by disrupting host tissue and sucking up blood that bleeds into the feeding site (Ribeiro, 1995; Francischetti et al., 2009). This feeding style activates host defense pathways that are aimed at stopping further blood loss. Ticks successfully feed by injecting hundreds of saliva proteins into the host to block host defense to tick feeding (Mudenda et al., 2014; Radulović et al., 2014; Tirloni et al., 2014, 2015; Kim et al., 2016b). Among the molecules present in tick saliva, those that modulate pain/itching, hemostasis, inflammation, wound healing, and host immunity are considered the most important in tick-host-pathogen interaction as these proteins allow blood meal acquisition and facilitate TBD pathogen transmission (Ribeiro, 1995; Nuttall and Labuda, 2004; Francischetti et al., 2009).

The profiles of proteins in tick saliva during blood feeding are different depending on the tick species and the stage of the tick (Mudenda et al., 2014; Radulović et al., 2014; Tirloni et al., 2014, 2015; Kim et al., 2016b). Whether or not ticks of the same species inject the same or different profiles of proteins when feeding on different animal hosts remain unknown. Resolving this question will be particularly interesting for ticks such as *A. americanum* and *I. scapularis* that feed on immunologically diverse animal species, from birds to large mammals (Keirans et al., 1996; Kollars et al., 2000), as the hemostatic and immune responses of their different hosts vary considerably (Gentry, 2004; Boehm, 2012). Furthermore, there is evidence that due repetitive infestations, ticks are able to induce a very strong resistance in some hosts species but not in others, suggesting that resistance is centered on host's particular immune characteristics and/or in the evolution of highly specific evasion mechanisms in ticks due saliva composition (Szabó and Bechara, 1999). In the same way, recently a study demonstrated that *I. scapularis* saliva displays variable fibrinogenolytic activities upon feeding on hosts with different immune backgrounds (Vora et al., 2017). Thus, it is reasonable to hypothesize that ticks could switch their salivary composition in order to modulate different host defense responses.

There is evidence that when ticks engage the host they express certain genes that are thought to represent the tick's molecular preparation to start feeding. Mulenga et al. (2007) described 40 transcripts that were differentially up regulated in *A. americanum* ticks that were stimulated to start feeding on cattle. Likewise, Lew-Tabor et al. (2010) and Rodriguez-Valle et al. (2010) identified differentially up-regulated genes in *R. microplus* that were stimulated to start feeding on cattle. In a related study, Popara et al. (2013) demonstrated differential protein expression in *R. microplus* that fed on cattle and white-tailed deer. Studies reviewed here (Mulenga et al., 2007; Lew-Tabor et al., 2010; Rodriguez-Valle et al., 2010; Popara et al., 2013) suggested that ticks may express specific genes to prepare for feeding on different host species. In this study, we provide evidence that protein profiles in saliva of ticks that are stimulated to start feeding on different change, as suggested by differential protein profiles in saliva of both *A. americanum* and *I. scapularis* ticks, which were stimulated to start feeding on different hosts.

## Materials and methods

### Ethics statement

Ticks used in this study were unfed adult females. As ticks were not fed, modifications of the host to feed ticks were not required, with exception of rabbits. For rabbits, we attached a cotton stockinet on top of the rabbit ear as outlined in the animal use protocol 2011-187 that was approved by Texas A&M University IACUC. The cotton stockinet attachment was used to contain the tick stimulation chamber as detailed below.

### Stimulating unfed adult *Ixodes scapularis* and *Amblyomma americanum* females to feed on different hosts

Adult *Ixodes scapularis* and *Amblyomma americanum* ticks that were used in this study were purchased from the tick rearing facilities at Oklahoma State University (Stillwater, OK, USA). Stimulation of unfed adult *A. americanum* and *I. scapularis* ticks to start feeding on different hosts: rabbits, dog, or human was done by exposing ticks to semio-chemicals and temperature as described (Mulenga et al., 2007) with modifications. The modification was that instead of a tick stimulation chamber being made out of a nylon mesh sachet, a tightly capped 2 mL vial that was perforated with a 27-gauge needle was used. The 27-gauge needle perforations were to allow semio-chemicals and body temperature to percolate ticks. Unfed *A. americanum* (*n* = 40 in each vial) and *I. scapularis* (*n* = 50–80 in each vial) females ticks were enclosed in a stimuli chamber and placed in close proximity with host skin. To ensure the cap did not open for ticks to escape during the stimulation step, the cap was secured with VWR® general-purpose laboratory labeling tape (VWR International, Radnor, PA, USA) and wrapped with Parafilm M® (Bemis Company Inc., Neenah, WI, USA). To expose ticks to semio-chemicals, the stimulation chamber containing ticks was placed in close proximity with host's skin for ~12 h. Three individual hosts: (i) human (*Homo sapiens*); (ii) New Zealand white rabbit (*Oryctolagus cuniculus*); and (iii) Dachshund dog (*Canis familiaris*) were used. To expose the ticks to human semio-chemicals and temperature, the stimuli chamber containing *A. americanum* or *I. scapularis* ticks was placed in the front shirt pocket of the volunteer. For exposure to rabbit, stimuli chambers were placed inside cotton stockinet that was attached onto the top of the rabbit ear (Kim et al., 2014). For dogs, stimuli chambers were taped on to the collar/harness. Following exposure to semio-chemicals, ticks were processed for saliva collection as describe below.

*I. scapularis* ticks mate off the host before interacting with the host (Sonenshine and Roe, 2014). From this perspective, *I. scapularis* female ticks were pre-mated before being stimulated to start feeding. This was done by putting female and male *I. scapularis* ticks in a container, and then visually identifying male and female pairs. Male and female *I. scapularis* pairs were placed in separate container to complete mating. Please note that, since *A. americanum* ticks mate after taking an initial blood meal (Sonenshine and Roe, 2014), we did not pre-mate females prior to stimulating them to start feeding.

### Tick saliva collection

Tick saliva was collected from unfed *I. scapularis* and *A. americanum* as previously described (Tirloni et al., 2014; Kim et al., 2016b). For *I. scapularis*, we collected saliva from unfed non-stimulated (*n* = 130 ticks) and those that were stimulated to feed on human (*n* = 80 ticks), rabbit (*n* = 130 ticks), and dog (*n* = 80 ticks) hosts. Likewise, for *A. americanum*, we collected saliva from non-stimulated (*n* = 40 ticks), and those that were stimulated to feed on human (*n* = 40 ticks), rabbit (*n* = 40 ticks), and dog (*n* = 40 ticks) hosts. Please note that non-stimulated ticks were taken from the incubator (22°C with 90% relative humidity) and were acclimated to room temperature during the saliva collection step. Ticks were rinsed in Milli-Q water, dried on a paper towel and placed dorsal-side down on a glass slide containing tape. Salivation was induced by injecting 0.5–1 μL of 2% pilocarpine hydrochloride (in PBS, pH 7.4) on the ventral side of the lower right coxa using a 34 gauge/0.5 inches/45° angle beveled needle on a model 701 Hamilton syringe (Hamilton Company, Reno, NV, USA). Tick saliva, which in some instances crystalized, was harvested in 2 μL of phosphate buffered saline (PBS) placed on tick mouthparts using a Hamilton syringe every 15 min over ~4 h at room temperature. Saliva protein concentrations were determined using BCA enhanced protocol (BCA Protein Assay, Pierce, Rockford, IL, USA). Tick saliva was lyophilized and stored at −80°C upon use.

### SDS-PAGE and silver staining

Approximately 1 μg of *I. scapularis* and 1.5 μg of *A. americanum* total saliva proteins were electrophoresed on 4–20% gradient SDS-PAGE. Gels were silver stained using the Pierce Silver Stain for Mass Spectrometry kit (Thermo Fisher Scientific, Waltham, MA, USA) according to manufacturer's instructions.

### Protein digestion and sample preparation

Total tick saliva proteins (2 μg, in triplicate) of *I. scapularis* or *A. americanum* ticks that were non-stimulated and those that were stimulated to starting feed on different hosts (human, dog, and rabbit) were diluted in 8 M urea/0.1 M Tris, pH 8.5, reduced with 5 mM Tris (2-carboxyethyl) phosphine hydrochloride (TCEP, Sigma-Aldrich, St Louis, MO, USA) and alkylated with 25 mM iodoacetamide (Sigma-Aldrich). Proteins were digested overnight at 37 °C in 2 M urea/0.1 M Tris pH 8.5, 1 mM CaCl_2_ with trypsin (Promega, Madison, WI, EUA) with a final ratio of 1:20 (enzyme:substrate). Digestion reactions, in a final concentration of 0.2 μg/mL, were quenched with formic acid (5% final concentration) and centrifuged for debris removal.

### Pre-columns and analytical columns

Reversed phase pre-columns were prepared in deactivated 250 μm ID/360 μm OD silica capillary (Agilent Technologies, Santa Clara, CA, USA) with a 2 mm Kasil frit at one end. Kasil frits were prepared by dipping 20 cm capillary in 300 μL Kasil 1624 (PQ Corporation, Malvern, PA, USA) and 100 μL formamide solution, curing at 100°C for 3 h and adjusting the length. Pre-columns were packed in-house with 2 cm of 5 μm ODS-AQ C18 (YMC America, INC., Allentown, PA, USA) particles from particle slurries in methanol. Analytical reversed phase columns were fabricated by pulling a 100 μm ID/360 μm OD silica capillary (Molex Polymicro Technologies™, Austin, TX, USA) to a 5 μm ID tip. The same packing material was packed until 20 cm directly behind the pulled tip. Reversed phase precolumns and analytical columns were connected using a zero-dead volume union (IDEX Corp., Upchurch Scientific, Oak Harbor, WA, USA).

### LC-MS/MS

Peptide mixtures were analyzed by nanoflow liquid chromatography mass spectrometry using an Easy NanoLC II and a Q Exactive mass spectrometer (Thermo Scientific, Waltham, MA, USA). Peptides eluted from the analytical column were electrosprayed directly into the mass spectrometer. Solutions A and B consisted of 5% acetonitrile/0.1% formic acid and 80% acetonitrile/0.1% formic acid, respectively. The flow rate was set to 400 nL/min. Saliva samples (2 μg per injection) were separated in 155-min chromatographic runs, as follows: 1–10% gradient of solution B in 10 min, 10–40% of solution B in 100 min, 40–50% of solution B in 10 min and 50–90% of solution B in 10 min. Column was held at 90% of solution B for 10 min, reduced to 1% of solution B and re-equilibrated prior to next injection.

The mass spectrometer was operated in a data dependent mode, collecting a full MS scan from 400 to 1,200 m/z at 70,000 resolution and an AGC target of 1 × 10^6^. The 10 most abundant ions per scan were selected for MS/MS at 17,500 resolution and AGC target of 2 × 10^5^ and an underfill ratio of 0.1%. Maximum fill times were 20 and 120 ms for MS and MS/MS scans, respectively, with dynamic exclusion of 15 s. Normalized collision energy was set to 25.

### Data analysis

Tandem mass spectra were extracted from Thermo RAW files using RawExtract 1.9.9.2 (McDonald et al., 2004) and searched with ProLuCID (Xu et al., 2015) against a non-redundant tick databases. *I. scapularis* peptides were searched against the database containing an Ixodidae sequences from NCBI (62,246 sequences and reverse sequences). For *A. americanum*, we searched peptides against an in house database of translated whole tick and dissected organ transcriptome (BioProject accession number PRJNA226980). Searches were done using Integrated Proteomics Pipeline—IP2 (Integrated Proteomics Applications, Inc.). The search space included all fully-tryptic and half-tryptic peptide candidates. Carbamidomethylation on cysteine was used as static modification. Data was searched with 50 ppm precursor ion tolerance and 20 ppm fragment ion tolerance.

The validity of the peptide spectrum matches (PSMs) generated by ProLuCID was assessed using Search Engine Processor (SEPro) module from PatternLab for Proteomics platform (Carvalho et al., 2015). Identifications were grouped by charge state and tryptic status, resulting in four distinct subgroups. For each group, ProLuCID XCorr, DeltaCN, DeltaMass, ZScore, number of peaks matched and secondary rank values were used to generate a Bayesian discriminating function. A cut-off score was established to accept a false discovery rate (FDR) of 1% based on the number of decoys. This procedure was independently performed on each data subset, resulting in a false-positive rate that was independent of tryptic status or charge state. Additionally, a minimum sequence length of six residues per peptide was required. Results were post-processed to only accept PSMs with <10 ppm precursor mass error. A Principal Component Analysis (PCA) plot, performed using PatternLab's Buzios module (Carvalho et al., 2015), was employed to aid in interpreting similarities among samples. Venn's four-set diagrams were generated from the output of PatternLab's Birds Eye view report. Proteins were grouped by maximum parsimony and the presence of proteins in at least two out of three replicates was required for each condition.

Volcano plots were generated by a pairwise comparison between non-stimulated and stimulated tick saliva using PatternLab's TFold module, which uses a theoretical FDR estimator to maximize identifications satisfying both a fold-change cut-off that varies with the *t*-test *p*-value as a power law and a stringency criterion that aims to fish out proteins of low abundance that are likely to have had their quantitation compromised (Carvalho et al., 2015). The following parameters were used to select differentially expressed proteins: proteins were grouped by maximum parsimony, spectral count data was normalized using normalized spectral abundance factor (NSAF) (Zybailov et al., 2006), two (out of the three runs) non-zero replicate values were required for each condition, and a BH *q*-value was set at 0.02 (2% FDR). Low abundant proteins were removed using an L-stringency value of 0.2.

### Functional annotation and classification

To get insight on the nature of the identified protein sequences, BLASTp searches against several databases were performed. To functionally classify the protein sequences, a program written and provided by Dr. José M. C. Ribeiro in Visual Basic 6.0 (Microsoft, Redmond, WA, USA) was used (Karim et al., 2011). The functionally annotated catalog for each dataset was manually curated and plotted in a hyperlinked Excel spreadsheet designed as Table S1 (for *I. scapularis*) and Table S2 (for *A. americanum*).

We have recently identified proteins in saliva of *I. scapularis* ticks that were fed every 24 h on rabbits (Kim et al., 2016b). To determine if some proteins reported here were injected into the host during tick feeding, unfed *I. scapularis* tick saliva proteome from this study were scanned against published *I. scapularis* proteome.

### Relative abundance and graphical visualization

Proteomic profiles were compared across samples as functional classes or individual proteins. To determine the relative abundance of proteins, normalized spectral abundance factor (NSAF) was used in a label-free relative quantification approach (Paoletti et al., 2006). Mean NSAF values from the two or three replicates were determined and combined according to functional class, and then divided by the total NSAF for the respective sample. NSAF as an index for relative protein abundance was input in Microsoft Excel (Microsoft, Redmond, WA, USA) as percentage of the total NSAF for respective samples, and visualized on pie charts according to protein classes. To visualize relative expression patterns on a heat map, NSAF values were normalized using Z-score. Normalized NSAF values were used to generate heat maps using the heatmap.2 function from the gplots package in R.

### Data availability

The mass spectrometry proteomics raw data have been deposited to the ProteomeXchange Consortium via the PRIDE partner repository with the dataset identifier PXD00712.

## Results and discussion

### Ticks stimulated to feed on different hosts have unique protein profiles in their saliva

**Figure 1:**
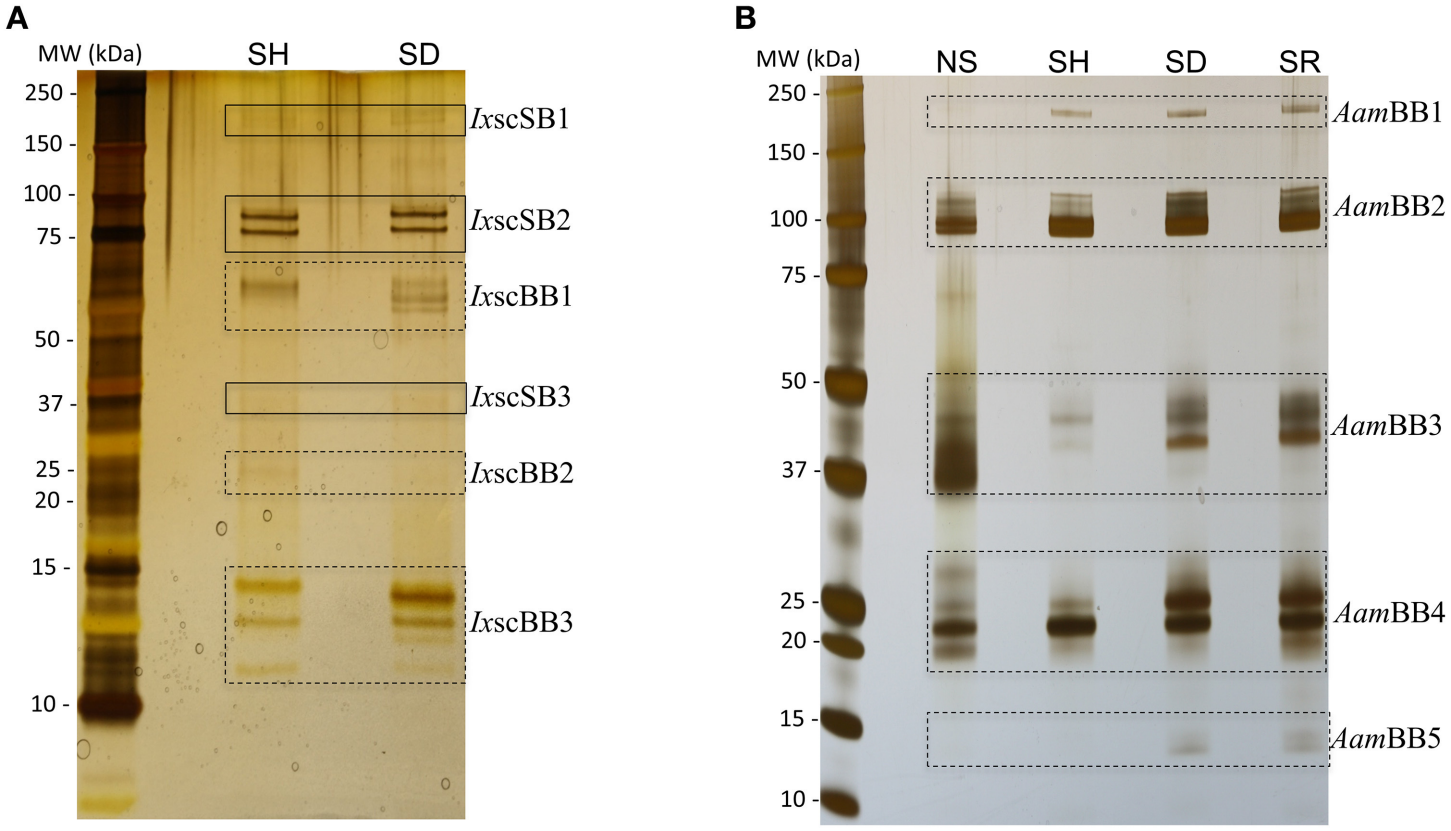
Stimulating ticks to start feeding on different hosts induces expression of differential tick-saliva proteins. To induce salivation, adult female unfed **(A)** *Ixodes scapularis* and **(B)** *Amblyomma americanum* ticks that were stimulated to feed on human (SH), dog (SD), rabbit (SR) for ~12 h and those not stimulated to start feeding (NS, were taken straight from the incubator) were microinjected with 0.5–1 μL of 2% pilocarpine hydrochloride. Approximately 1 μg of *I. scapularis* and 1.5 μg of *A. americanum* total saliva proteins was electrophoresed on 4–20% SDS gradient gels and silver stained. Solid (SB) and broken (BB) line boxes respectively highlight apparently identical and different protein banding patterns in saliva of *I. scapularis* (*Ixsc*) and *A. americanum* (*Aam*) exposed to different hosts. Please note that due to insufficient samples, we did not run *I. scapularis* NS and SR samples.

The hard tick's adaptation to feed on different animal hosts is central to TBD epidemiology as it facilitates the tick's movement of TBD agents from their wild animal reservoir hosts to humans, farm and companion animals. Despite its centrality, the molecular basis of how the tick adapts to feed on different hosts has not been fully evaluated. The tick feeding style of lacerating host tissue and then sucking up host blood that bleeds into the wounded area (commonly known as the tick-feeding site) is thought to stimulate host tissue repair responses that are aimed at stopping further blood loss. However, ticks ensure a full blood meal by secreting a cocktail of proteins that disarm the host's tissue repair response (Francischetti et al., 2009). There is also evidence the tick may express specific sets of genes in preparation to start feeding (Mulenga et al., 2007; Lew-Tabor et al., 2010; Rodriguez-Valle et al., 2010). This study was initiated to answer the question of whether or not the molecular preparation to start feeding by ticks such as *I. scapularis* and *A. americanum* that are adapted to feed on a wide range of hosts utilized was the same regardless of the host. Findings in this study suggest that the tick's molecular preparation to start feeding could be host-specific as indicated by SDS-PAGE and silver staining analysis that revealed unique protein profiles in saliva of *I. scapularis* and *A. americanum* ticks that were stimulated to start feeding on rabbits (SR), dog (SD), human (SH), and those that were not-stimulated (NS) (Figure 1). Solid (SB) and broken (BB) line boxes respectively highlight similarity and differences of detectable protein band patterns in saliva of ticks that were stimulated to feed on different hosts (Figure 1). Whereas we observed similarities (solid boxes) and differences (broken boxes) in protein banding patterns in saliva of *I. scapularis* ticks that were stimulated to feed on dogs and humans (Figure 1A), there are no apparent similarities among protein banding patterns in saliva of the four *A. americanum* treatments (Figure 1B). A notable observation in Figure 1B is that protein banding patterns in saliva of dog and rabbit-stimulated *A. americanum* ticks were closely similar, while those exposed to humans show more differences. Due to insufficient sample amounts, non-stimulated and rabbit-exposed *I. scapularis* tick saliva proteins were not analyzed by SDS-PAGE (Figure 1A). From this perspective, our SDS-PAGE analysis in Figure 1A was limited, and thus its unclear if observations in *A. americanum* were consistent with those in *I. scapularis*. The observation that the protein profile in saliva of non-stimulated *A. americanum* ticks is different from those that were stimulated to start feeding (Figure 1B) further demonstrates that stimulating ticks to start feeding impacted proteins that were secreted into tick saliva.

There is evidence that exposing the animal to high temperature stress can affect expression levels of different proteins (Villar et al., 2010). However, given that the respective axillary temperatures of dogs (~38°C), human (~37°C), and rabbits (~36°C) are not dramatically different (Vadlejch et al., 2010; Goic et al., 2014), it is unlikely that the observed differences in protein profiles were temperature dependent. For our non-stimulated tick cohorts, saliva was collected from ticks that were obtained straight from the incubator at 22°C and acclimated to room temperature, which is normally around 25°C. Thus, observed shift in protein profiles between NS and those that were stimulated to start feeding on different hosts (which have higher respective axillary temperatures) cannot be ruled out. On the other hand, studies have been shown that ticks respond to mechano-, chemo-, and thermo-sensation and are able to induce different electrophysiological responses (Soares and Borges, 2012; Sonenshine and Roe, 2014). Upon contact with host, release of host-derived semio-chemicals and their interaction with tick sensory organs may result in different electrophysiological responses leading secretion of different proteins during host stimulation process. Since ticks used in this study did not come into contact with the host's skin, tick saliva proteins described reported may not include those are responsive to mechano-sensory stimulation.

**Figure 2:**
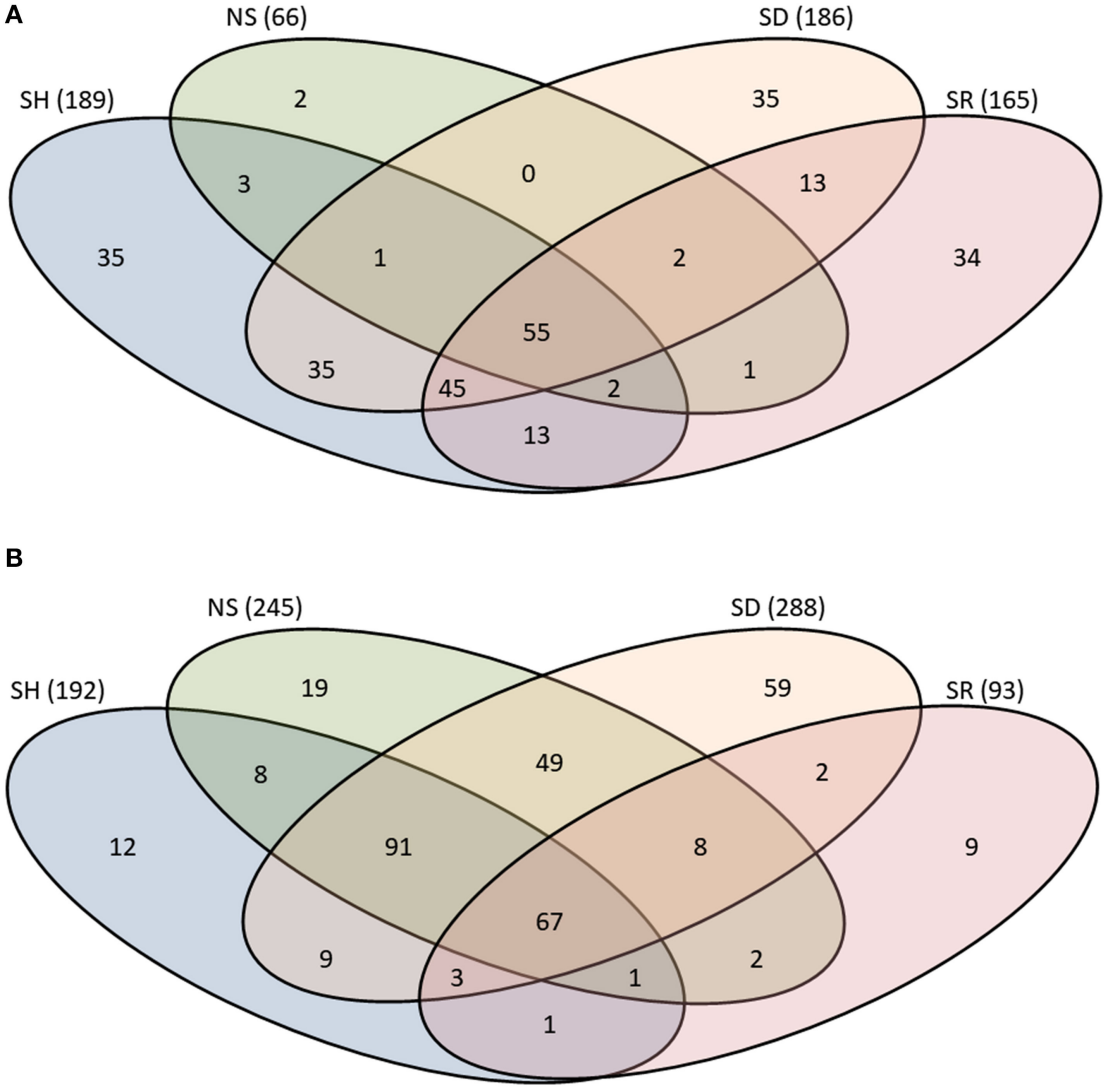
Protein counts in saliva of *Ixodes scapularis* **(A)** and *Amblyomma americanum* **(B)** ticks that were not-stimulated (NS) to start feeding, and those that were stimulated to feed on rabbits (SR), dog (SD), and human (SH). Total number of proteins for each treatment is indicated in parenthesis. The overlap region between Venn diagrams shows proteins present in two or more treatments.

**Figure 3:**
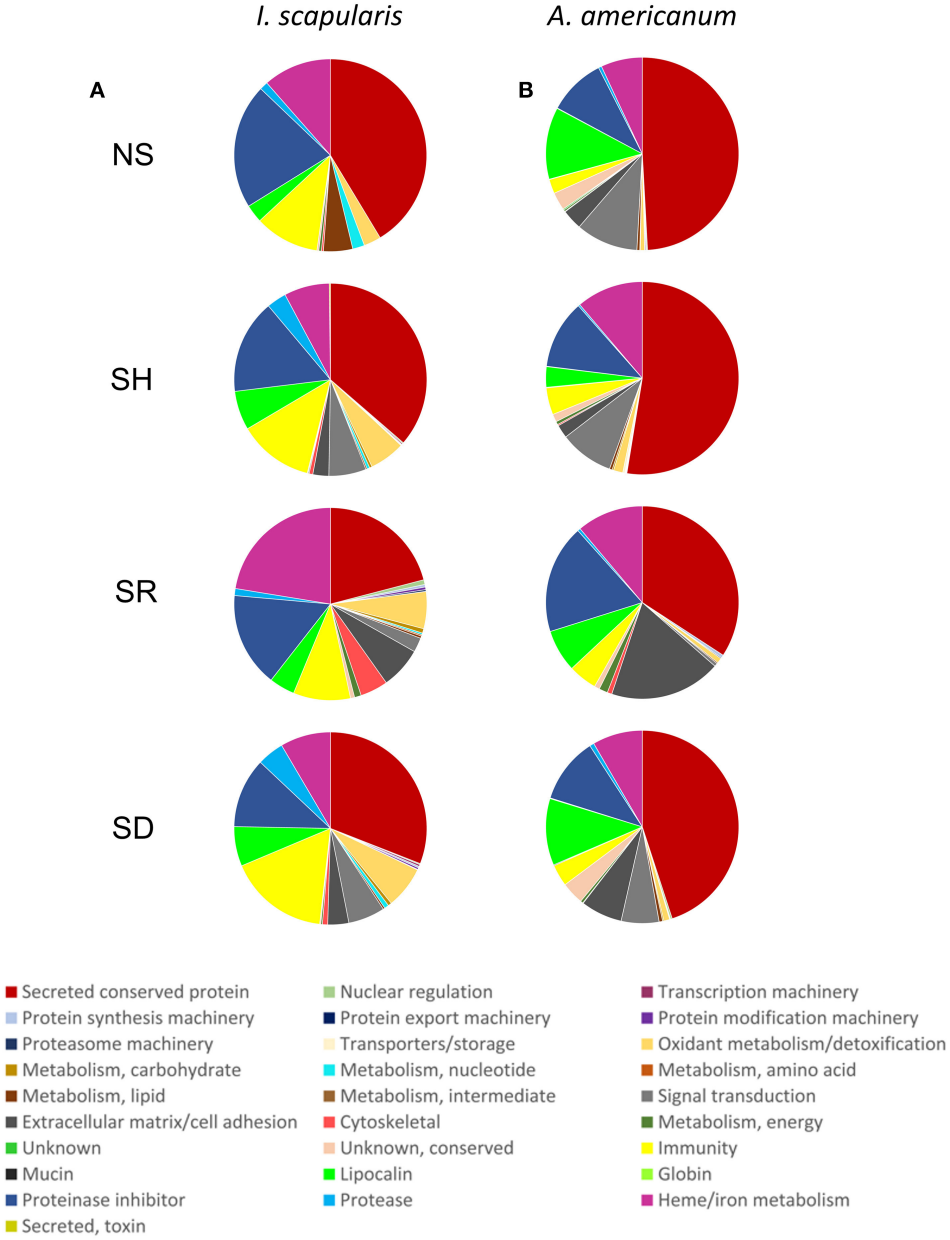
Relative abundance of protein functional classes in saliva of *Ixodes scapularis* **(A)** and *Amblyomma americanum* **(B)** ticks that were not-stimulated (NS) to start feeding, and those that were stimulated to feed on human (SH), rabbit (SR), and dog (SD). The sum of normalized spectral abundance factor (NSAF) for each functional class is represented as the percentage of total NSAF.

To get further insights into differences observed among saliva proteomes from ticks that were exposed to different hosts, we proceeded to identify proteins by LC-MS/MS using an *in-solution* digestion approach as described (Tirloni et al., 2014; Kim et al., 2016b). We respectively identified a total of 276 and 340 non-redundant *I. scapularis* (Figure 2A) and *A. americanum* (Figure 2B) tick saliva proteins that were determined authentic as they were detected in two or all of the three LC-MS/MS runs that were done for each sample (Figure 2, Tables S1, S2). The remaining 69 (*I. scapularis*) and 57 (*A. americanum*) proteins that were detected in only one of the three runs were considered low confidence hits and not further discussed (Tables S1, S2). Figure 2 summarizes the overall total proteins that were identified in saliva of ticks not stimulated to start feeding (NS) and those stimulated to start feeding on human (SH), dog (SD), and rabbit (SR). Of the 276 unique *I. scapularis* tick saliva proteins (Table S1), 66, 189, 186, and 165 were respectively identified in saliva of NS, SH, SD, and SR ticks (Figure 2A). Of these, 55 were common to all treatments, while 2, 35, 35, and 34 proteins were respectively unique to NS, SH, SD, and SR ticks (Figure 2A). Likewise, in *A. americanum* we respectively found 245, 192, 288, and 93 proteins in saliva of NS, SH, SD, and SR ticks (Figure 2B). Of these, 67 were common to all treatments, while 19, 12, 59, and 9 proteins were respectively unique to NS, SH, SD, and SR ticks (Figure 2B). Functional annotations classified both *I. scapularis* and *A. americanum* tick saliva proteins into 28 protein classes (Figure 3, Tables S1, S2). Based on the total sum of normalized spectral abundance factor (NSAF) (Figure 3), the predominant classes of proteins in this study include secreted conserved proteins (unknown functions), proteinase inhibitors, lipocalins, extracellular matrix/cell adhesion, heme/iron metabolism, signal transduction and immunity-related proteins (Figure 3).

**Figure 4:**
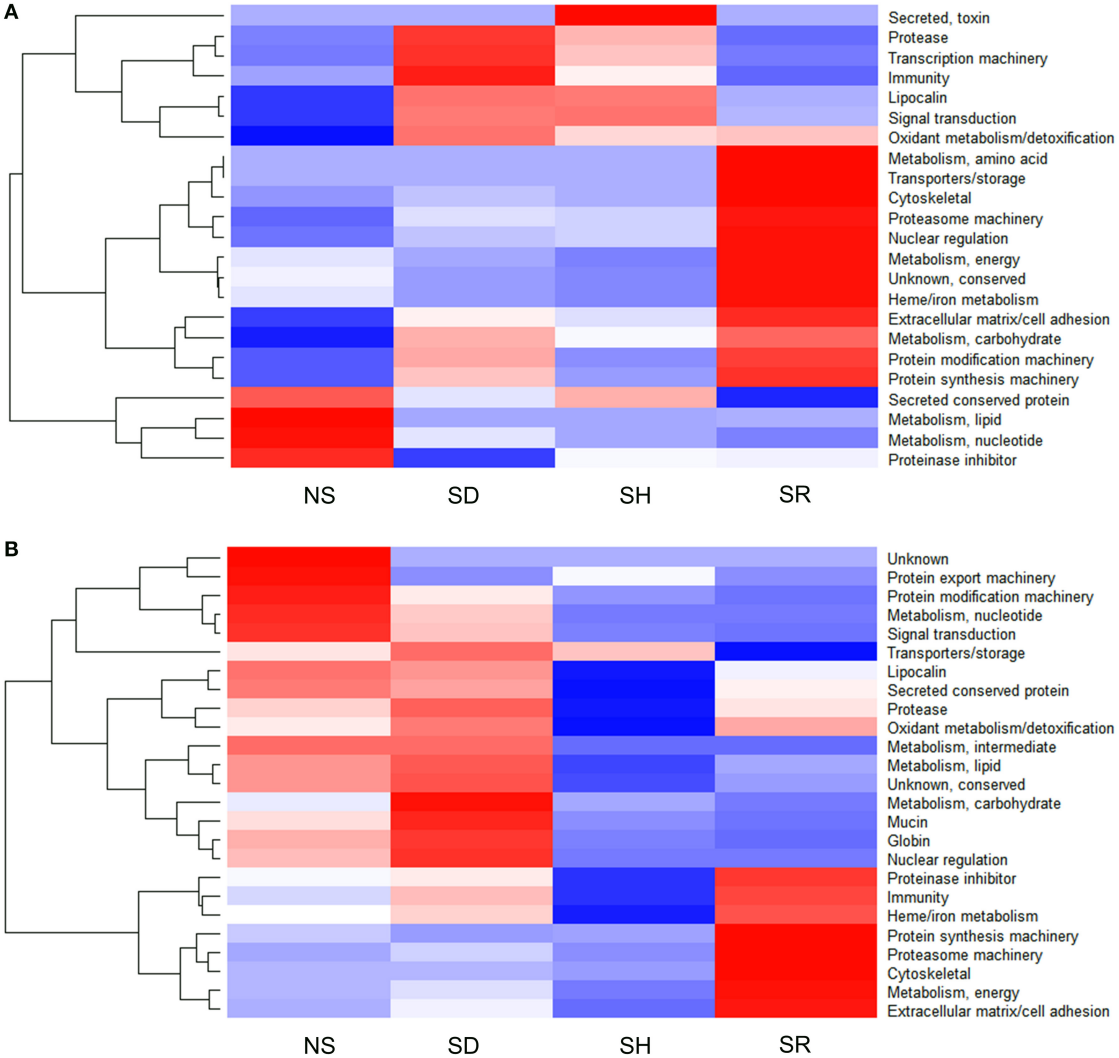
Proteins in saliva of *Ixodes scapularis* **(A)** and *Amblyomma americanum* **(B)** ticks that were not-stimulated (NS) to start feeding, and those that were stimulated to feed on dog (SD), human (SH), and rabbit (SR) are differentially abundant. Normalized spectral abundance factor (NSAF) for each protein is expressed as a percent of total NSAF. Z-scores were calculated and used to generate heat maps as described in Materials and Methods section. Red color indicates proteins of high abundance and blue color indicates proteins of low abundance.

Graphic visualization data in Figure 4 summarizes the Z-score of normalized NSAF values for each of the 28 functional classes identified in *I. scapularis* (Figure 4A) and *A. americanum* (Figure 4B). These data reveal two general trends: (i) the tick might inject the same protein at different levels into different hosts, and that (ii) protein composition in saliva of different tick species that feed on the same host is likely different (Figure 4). For instance, saliva of SR *I. scapularis* ticks had high abundance of heme/iron metabolism (22.4%) followed by extracellular matrix/cell adhesion (7%), oxidant metabolism/detoxification (6.2%), cytoskeletal (4.6%), metabolism (amino acid, carbohydrate and energy) (2%), proteasome machinery (1.1%), nuclear regulation (0.8%), conserved protein with unknown functions (0.6%), protein modification (0.4%), protein synthesis machinery proteins (0.4%), and transport/storage (0.02%) (Figure 4A and Table S1). In contrast, saliva of SR *A. americanum* ticks had high abundance of extracellular matrix/cell adhesion (18.6%) and proteinase inhibitors (18.2%), followed by heme/iron metabolism (11.1%), immunity-related (4.8%), metabolism of energy proteins (1.4%), cytoskeletal (0.7%), protein synthesis machinery (0.5%), and proteasome machinery (0.1%) (Figure 4B and Table S2).

### *I. scapularis* and *A. americanum* ticks stimulated to feed on different hosts secrete a core set of functionally similar proteins in their saliva

An interesting finding in our data is that the 55 and 67 proteins that were respectively found in all *I. scapularis* and *A. americanum* treatments (Figures 2A,B) belonged to the same functional classes (Figure S1, Tables S1, S2). These data suggest that *A. americanum* and *I. scapularis* utilized a core set of functionally similar proteins that regulated key host defense pathways to successfully feed. Although functional role(s) of proteins in Figure S1 remain to be determined, available evidence indicate that some of these proteins regulated important tick feeding pathways. For instance, a cross-tick species conserved AV422 protein that was originally identified among genes that were up regulated in *A. americanum* ticks that were stimulated to feed on cattle (Mulenga et al., 2007) and was injected into animals during *A. americanum* (Mulenga et al., 2013), *I. scapularis* (Kim et al., 2016b), *R. microplus* (Tirloni et al., 2014), and *Haemaphysalis longicornis* (Tirloni et al., 2015) feeding is an inhibitor of blood clotting and platelet aggregation (Mulenga et al., 2013). Likewise, EEC19556.1, which was found in all *I. scapularis* treatments (Table S1) is 99% identical to a serine protease inhibitor (serpin, AID54718.1) anti-coagulant and inhibitor of thrombin (Ibelli et al., 2014) that is injected into rabbits during *I. scapularis* feeding (Kim et al., 2016b).

### Proteins in saliva of ticks stimulated to feed on different hosts are differentially abundant

**Figure 5:**
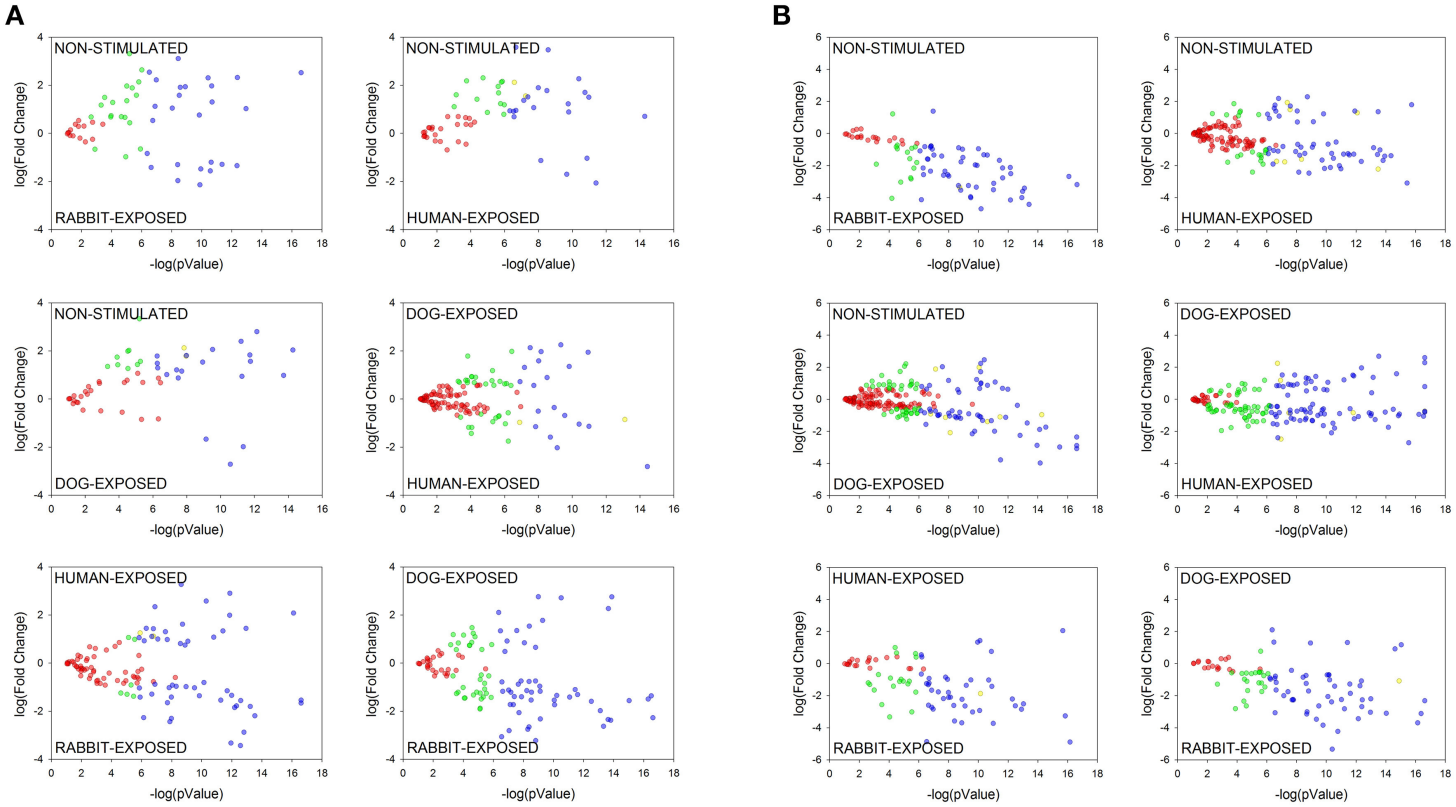
Pairwise comparative analysis showing differentially abundant proteins in saliva of *Ixodes scapularis* **(A)** and *Amblyomma americanum* **(B)** ticks stimulated to feed on different hosts. Normalized spectral abundance factor (NSAF) for each protein was subjected to pairwise analysis as detailed in section Materials and Methods. Each protein is represented as a dot and is mapped according to its fold change on the ordinate axis (y) and *t-*test *p*-value on the abscissa axis (x). Proteins represented by: (i) blue dots are significantly enhanced, (ii) yellow dots are significantly different but not satisfied the fold criteria, (iii) green dots satisfied the fold criteria but not significantly different, (iv) red dots not significantly different (present at equal abundance in both treatments). Details of the computational comparison are available in Tables S4, S5.

In order to investigate if shared proteins were differentially secreted *I. scapularis* and *A. americanum* when stimulated to start feeding on different hosts, pairwise comparison analyses using the PatternLab's TFold module (Carvalho et al., 2015) were conducted (Figure 5, Tables S4, S5). This analysis demonstrated that some of the shared proteins were secreted at equivalent levels (red dots), not significantly different (green and yellow dots), and significantly at different levels (blue dots) (Figure 5). Based on fold change (FC), differences in abundance ranged between 18.0 and 1.2 for *I. scapularis* (Table S4) and between 40 and 1.2 for *A. americanum* (Table S5). Consistent with our analysis in Figure 4, majority of SR *I. scapularis* tick saliva proteins were secreted at high concentrations when compared to either SD or SH. When compared to SD or SH, some of the most abundant proteins in saliva of SR *I. scapularis* ticks, included superoxide dismutase (EEC10196.1, FC: 17.7, and FC: 9.3), a glyceraldehyde-3-phosphate dehydrogenase—GAPDH (JAA68969.1, FC: 10.7), a tropomyosin (JAB83342.1, FC: 9.9), a thymosin (JAA70823.1, FC: 7.3), a fructose 1,6-biphosphate aldolase (EEC14101.1, FC: 8.3), and a creatine kinase (JAB78095.1, FC: 6.9). When paired with either rabbit or human, proteins with higher FC in saliva of SD *I. scapularis* ticks, include oxidase/peroxidase enzyme (EEC07462.1, FC: 18.0), a serine protease (EEC02857.1, FC: 6.7), a secreted salivary gland peptide (EEC14213.1, FC: 6.7), a peroxinectin (EEC08358.1, FC: 6.5), a microplusin (AAY66495.1, FC: 4.8), a GAPDH (JAA68969.1, FC: 4.7), and a 14-3-3 zeta protein (JAB76832.1, FC: 4.3). Likewise, for human-exposed ticks an oxidase/peroxidase (EEC07462.1, FC: 9.6), a secreted salivary gland peptide (EEC14213.1, FC: 7.5), an insulin growth factor-binding protein (EEC07853.1, FC: 5.0), a metalloprotease (AAM93652.1, FC: 7.0), and a secreted protein (EEC14470.1, FC: 4.0) were identified with higher FC (Table S4).

Although similar to *I. scapularis* (Table S4), proteins in saliva of *A. americanum* were likely secreted at high concentration when stimulated to feed on rabbits than either dog or human (Table S5). It is notable that fewer proteins were differentially abundant in SH (*n* = 2) or SD (*n* = 4) *A. americanum* tick saliva when compared with SR saliva (Figure 5B and Table S5). Please note that four of the six proteins that were differentially abundant in SH compared to SR *A. americanum* saliva (Figure 5B) are potentially contaminating keratins (Table S5), and hence the six blue dots in Figure 5. Whether or not the observation here is a consistent biological phenomenon remains to be determined. We also think that this observation could be explained by the limitation of our study: we searched *A. americanum* peptides against a database of a translated *A. americanum* transcriptome that was generated from ticks that were fed on rabbits. We anticipate that if the *A. americanum* genome became available, additional host-dependent *A. americanum* tick saliva protein secretions will be identified.

Despite the fact that we identified a limited number of differentially abundant *A. americanum* proteins, the secretion dynamics of AV422 (Mulenga et al., 2007) was similar in both *A. americanum* and *I. scapularis* (Tables S4, S5). Our data here show this protein is secreted at high abundance when *A. americanum* is stimulated to feed on humans (FC: 1.7) and dogs (FC: 2.2). Similarly, in *I. scapularis*, AV422 homolog is secreted at high abundance when *I. scapularis* is stimulated to feed on humans (FC: 1.6) and dogs (FC: 1.8). It will be interesting to further investigate the role(s) of *I. scapularis* AV422 homolog in tick feeding were similar to those observed *A. americanum* (Mulenga et al., 2013).

The observation that, *A. americanum* ticks may secrete fatty acid-binding protein at FC of 40.2 (Aam-134, Table S5) represents the most abundantly secreted protein in saliva of SR *A. americanum* tick saliva. This protein was identified exclusively in saliva of SR and SD ticks and appears more abundant in the former (Table S5). Fatty acid-binding proteins have been identified in helminth secretions (Morphew et al., 2007) and in tick saliva. This protein has been described to modulate human monocyte-derived macrophages and generate M2 macrophages (MΦ) activated by the alternative pathway (Figueroa-Santiago and Espino, 2014). M2 MΦ are characterized by secretion of anti-inflammatory cytokines (Gordon, 2003). Since ticks have to evade the host's inflammation defense, could the fatty acid-binding protein being among tick proteins that modulate host defense? It would be interesting to address if the *A. americanum* fatty acid-binding protein has similar effects on host immune modulation. Other proteins showing high FC values in saliva of *A. americanum* SR ticks are glycine-rich proteins: Aam-178421 (FC: 29.1), Aam-1227 (FC: 29.0), Aam-327 (FC: 13.1) when pairing with SH; and Aam-179267 (FC: 18.6), Aam-177922 (FC: 14.2), and Aam178421 (FC: 12.8) when pairing with SD (Table S5). Glycine-rich proteins are extracellular matrix proteins and/or structural proteins thought to play an important role in attachment to the host, as they are present in cement material secreted by salivary glands during feeding process (Bishop et al., 2002; Maruyama et al., 2010). Since these proteins are secreted in the early stage of tick feeding, we suggest that glycine-rich proteins could be associated with tick cement formation, securely anchoring ticks onto host skin during the prolonged tick-feeding period. Another set of proteins with high FC values include a hemelipoprotein (FC: 21.3), a GAPDH (FC: 16.0), several serpins (FC: 15.9 to 1.5), among others (Table S5). More details about the computational comparison are available in Figure 5, Tables S4, S5.

The observation of apparent differential expression is not likely peculiar to this study. In a lone study, tick proteins involved in blood digestion and reproduction were overrepresented in *R. microplus* ticks that fed on cattle when compared to ticks that fed on white-tailed deer (Popara et al., 2013). In a related study, *I. scapularis* displayed variable fibrinogenolytic activities upon feeding on mice with different immune backgrounds (Vora et al., 2017).

## Conclusions and future perspectives

It is important to note that proteins being discussed here were identified in saliva of unfed adult ticks. Findings that 83 of the 165 proteins found in SR *I. scapularis* tick saliva were also identified in the saliva of *I. scapularis* ticks that were fed on rabbits (Table S3) gives us confidence these proteins are also injected into the host during feeding. Of these 83 unique SR tick saliva proteins, 52, 67, 50, 58, 38, 37, and 59 proteins (Table S3) were respectively found in saliva of *I. scapularis* that fed on rabbits for 24, 48, 72, 96, and 120 h of feeding as well as those that were engorged but not detached and those that repelete fed and detached (Kim et al., 2016b). Of significance, 47 of the 55 *I. scapularis* proteins that were found in all treatments (Figure 2A) were also identified in saliva of this tick during feeding (Kim et al., 2016b; Tables S1, S3). While, it is apparent that some of the proteins that we found in saliva of SR *I. scapularis* ticks, we cannot confirm at this point if proteins in saliva of unfed SD and SH *I. scapularis* ticks are secreted during feeding as we cannot ethically feed ticks on humans for research purposes. This same limitation applies to our *A. americanum* tick saliva proteins in this study. We may be able to confirm in dogs, however it will be difficult to prove for humans. Despite these limitations, data here provides the first step toward the molecular basis of host range adaptation. To our knowledge, this work is the first report describing the use of LC-MS/MS analysis aimed at addressing the biologically relevant question of tick saliva plasticity when ticks are stimulated to feed on different hosts. Our data suggest that the tick's molecular preparation to start feeding is likely host-specific, as by differential protein profiles in saliva of both *A. americanum* and *I. scapularis* ticks which were stimulated to start feeding on different hosts. Within these different protein profiles there is a set of proteins that the tick may utilize to feed on all hosts. From the perspective of development of vaccines against tick feeding, data here has practical to the field of the molecular basis of tick feeding physiology, and tick vaccine development in particular. With the exception of anti-*R. microplus* vaccine research, for which its natural host, cattle are used for feeding, various laboratory animals such as lagomorphs and rodents are used as hosts for tick feeding, despite introduction of techniques involving artificial feeding on either animal skins or synthetic membranes (Waladde et al., 1991; Gonsioroski et al., 2012; Hatta et al., 2012). Therefore, contemporary research to develop vaccines against medically important tick species utilizes laboratory animal models in initial screening to identify putative effective antigens (Sugino et al., 2003; de la Fuente and Kocan, 2006; Rodríguez-Mallon et al., 2012; Kim et al., 2016a). Our data here clearly demonstrates that there are potential flaws to the use of laboratory animals to identify putative anti-tick vaccine antigens. Given that the tick might inject different sets of proteins in different hosts, there is potential to focus on irrelevant proteins when model animals are used in initial screens. The core of proteins that the tick might inject into all hosts (Tables S1, S2 and Figure S1) or those that the tick might utilize to regulate feeding on both laboratory animal hosts such as rabbits and relevant hosts such as human and dogs (Figure 5) could be prioritized for tick vaccine development. New Zealand white rabbits are usually the most accessible and most suitable hosts that are routinely used in tick vaccine research (Troughton and Levin, 2007). However, often there are experimental results obtained using a laboratory model that do not match reality when applied in wildlife animals (Olds et al., 2016). This could potentially be a consequence of targeting proteins that are important to tick feeding success on a lab animal model, but not the relevant in another host. Therefore, the identification of saliva proteins that are secreted in different hosts, including laboratory models such as rabbits, will remove the risk of targeting irrelevant proteins.

## Author contributions

Conceived and designed the experiments: LT, TK, and AM. Performed the experiments: LT, TK, and AP, AM. Contributed reagents, materials, analysis tools: AM and JY. Drafting the article: LT, TK, AP, JY, IdS, and AM. Critical revision of the article: LT, TK, AP, JY, IdS, and AM.

### Conflict of interest statement

The authors declare that the research was conducted in the absence of any commercial or financial relationships that could be construed as a potential conflict of interest.

## Supplementary material

Figure S1 Protein counts in saliva of *Ixodes scapularis* and *Amblyomma americanum* ticks stimulated to feed on different hosts showing a core set of functionally similar proteins in their saliva. The sum of proteins for each functional class is represented as the percentage of total proteins identified. Details of protein identification are available in Tables S1, S2.

Table S1 *Ixodes scapularis* saliva proteins identified by LC-MS/MS.

Table S2 *Amblyomma americanum* saliva proteins identified by LC-MS/MS.

Table S3 Comparison of *Ixodes scapularis* tick saliva proteins identified upon stimulation to feed on different hosts and *Ixodes scapularis* tick saliva proteins secreted every 24 h during blood feeding.

Table S4 Differentially expressed proteins based on *t*-fold analyses comparing *Ixodes scapularis* saliva proteins from tick stimulated to feed on different hosts.

Table S5 Differentially expressed proteins based on *t*-fold analyses comparing *Amblyomma americanum* saliva proteins from tick stimulated to feed on different hosts.
