# Supplementary figures and images for: Tick-Host Range Adaptation: Changes in Protein Profiles in Unfed Adult Ixodes scapularis and Amblyomma americanum Saliva Stimulated to Feed on Different Hosts
Source: Front Cell Infect Microbiol. 2017 Dec 19;7:517. doi: 10.3389/fcimb.2017.00517 (PMC5742094; doi:10.3389/fcimb.2017.00517)

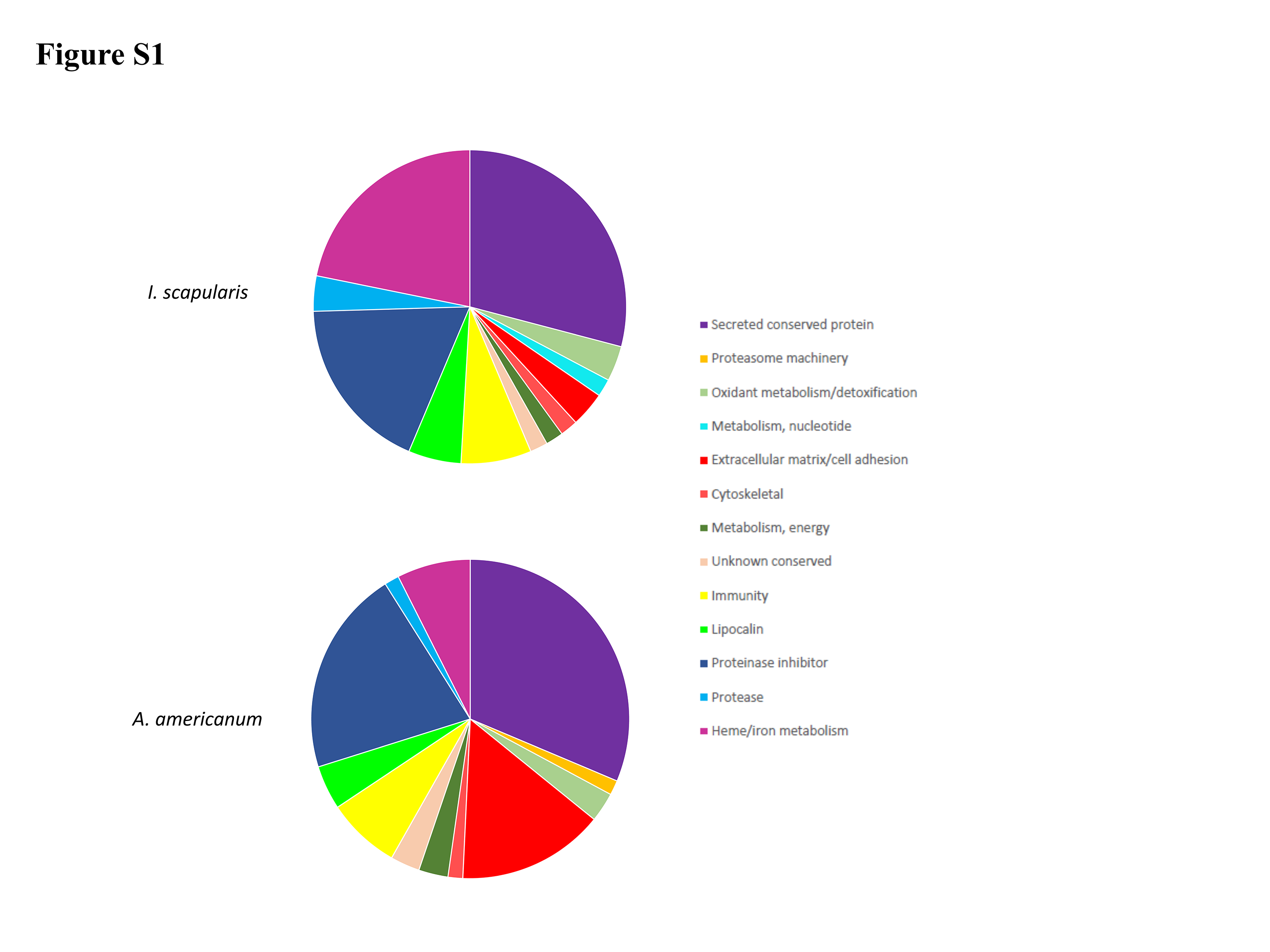

Supplement: Figure S1 — Protein counts in saliva of Ixodes scapularis and Amblyomma americanum ticks stimulated to feed on different hosts showing a core set of functionally similar proteins in their saliva. The sum of proteins for each functional class is represented as the percentage of total proteins identified. Details of protein identification are available in Tables S1, S2. [file Image1.TIF]
